# Supplementary material for: Methane seep carbonates yield clumped isotope signatures out of equilibrium with formation temperatures
Source: Nat Commun. 2016 Jul 22;7:12274. doi: 10.1038/ncomms12274 (PMC4961868; doi:10.1038/ncomms12274)
Supplement: Supplementary Information — Supplementary Figure 1, Supplementary Table 1 and Supplementary References [file ncomms12274-s1.pdf]

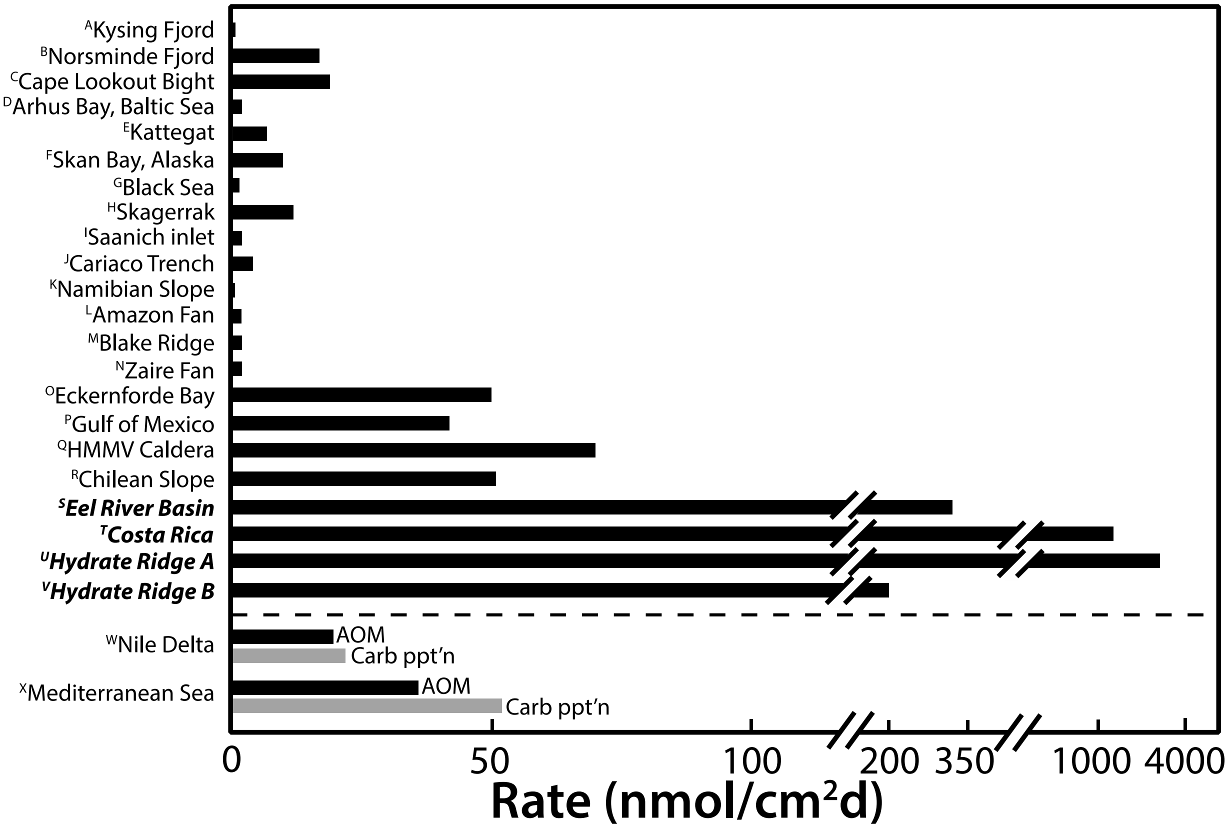

Supplementary Figure 1: AOM and carbonate precipitation rates of modern cold seep systems. Notice that where available, AOM rates overlap with carbonate precipitation rates. This association along with the relatively high AOM rates of Hydrate Ridge, Costa Rica and Eel River Basin suggest that these locations have a high propensity to record disequilibrium clumped isotope signatures. Data references as follows A[1]; B[2]; C[3]; D[4]; E[1]; F[5,6]; G[7]; H[1]; I[8]; J[9]; K[10,11]; L[12]; M[13]; N[14]; O[15]; P[16]; Q[17]; R[18]; S[19]; T[20]; U[21,22]; V[23]; W[24,25]; X[26,27].

Supplementary Table 1

Apparent ancient seep carbonate precipitation temperatures

Notice consistently high temperatures compared to typical bottom waters using both calibrations[28,29].

Sample	phases	temp (°C)	temp (°C)	s.e.
		[28]Tang et al., 2014	[29]Tripati et al., 2015	(°C)
Teepee Buttes				
100-110	micrite	134	97	5
130-140	micrite	193	128	5
190-200	micrite	160	112	7
20-30	micrite	163	113	3
230-240	micrite	207	135	4
250-260	micrite	260	159	6
310-320	micrite	286	169	8
320-2h	micrite	107	81	10
360-370	micrite	181	123	3
40-50	micrite	182	123	4
65-70	micrite	142	102	1
star 230-240	micrite	150	106	4
teepee hrm 210-220	micrite	185	125	5
teepee hrm 400-40	micrite	132	96	3
100-110fc	fibrous cement	178	121	3
190-200fc	fibrous cement	219	141	7
210-220fc	fibrous cement	263	160	9
460-470fc	fibrous cement	196	130	4
711.5 VI S 2	spar			
320-2 PS	peloidal sparite			
704.5 1 PS	peloidal sparite			
711 WI PS	peloidal sparite			
711.5 VI PS 2	peloidal sparite			
711.5 VI PS1	peloidal sparite	113	84	11
HRS 674.6 PS	peloidal sparite			
Quinault Fm				
Sample 1 Quin Fm	micrite	83	66	3
Sample 9 Quin	micrite	46	40	9
Sample 11 Quin Fm	micrite	42	37	2
Pysht Fm				
Sample 4 Pysht	micrite	47	41	7
Sample 5 Pysht	micrite	56	47	10
Panoche Hills				
PTH 04	micrite	28	27	2
PTH-07	micrite	19	20	2
PTH-08	micrite	27	26	8
PTH-18	micrite	31	29	1
PTH-19	micrite	40	35	1
Lincoln Creek Fm				
Sample 8 LC	micrite	46	40	7

References

1 Iversen, N. & Jorgensen, B. B. Anaerobic methane oxidation rates at the sulfate-methane transition in marine sediments from Kattegat and Skagerrak (Denmark) 1. *Limnology and Oceanography* **30**, 944-955 (1985).

2 Hansen, L. B., Finster, K., Fossing, H. & Iversen, N. Anaerobic methane oxidation in sulfate depleted sediments: effects of sulfate and molybdate additions. *Aquatic Microbial Ecology* **14**, 195-204 (1998).

3 Hoehler, T. M., Alperin, M. J., Albert, D. B. & Martens, C. S. Field and laboratory studies of methane oxidation in an anoxic marine sediment: Evidence for a methanogen-sulfate reducer consortium. *Global Biogeochemical Cycles* **8**, 451-463 (1994).

4 Thomsen, T. R., Finster, K. & Ramsing, N. B. Biogeochemical and molecular signatures of anaerobic methane oxidation in a marine sediment. *Applied and Environmental Microbiology* **67**, 1646-1656 (2001).

5 Reeburgh, W. S. Anaerobic methane oxidation: rate depth distributions in Skan Bay sediments. *Earth and Planetary Science Letters* **47**, 345-352 (1980).

6 Alperin, M. J. & Reeburgh, W. S. Inhibition experiments on anaerobic methane oxidation. *Applied and Environmental Microbiology* **50**, 940-945 (1985).

7 Jørgensen, B. B., Weber, A. & Zopfi, J. Sulfate reduction and anaerobic methane oxidation in Black Sea sediments. *Deep Sea Research Part I: Oceanographic Research Papers* **48**, 2097-2120 (2001).

8 Devol, A. H. Methane oxidation rates in the anaerobic sediments of Saanich Inlet1. *Limnology and Oceanography* **28**, 738-742 (1983).

9 Reeburgh, W. S. Methane consumption in Cariaco Trench waters and sediments. *Earth and Planetary Science Letters* **28**, 337-344 (1976).

10 Niewöhner, C., Hensen, C., Kasten, S., Zabel, M. & Schulz, H. Deep sulfate reduction completely mediated by anaerobic methane oxidation in sediments of the upwelling area off Namibia. *Geochimica et Cosmochimica Acta* **62**, 455-464 (1998).

11 Fossing, H., Ferdelman, T. G. & Berg, P. Sulfate reduction and methane oxidation in continental margin sediments influenced by irrigation (South-East Atlantic off Namibia). *Geochimica et Cosmochimica Acta* **64**, 897-910 (2000).

12 Burns, S. J. Carbon isotopic evidence for coupled sulfate reduction-methane oxidation in Amazon Fan sediments. *Geochimica et cosmochimica acta* **62**, 797-804 (1998).

13 Borowski, W. S., Hoehler, T. M., Alperin, M. J., Rodriguez, N. M. & Paull, C. K. in *Proceedings of the ocean drilling program, scientific results.* 87-99.

14 Zabel, M. & Schulz, H. D. Importance of submarine landslides for non-steady state conditions in pore water systems—lower Zaire (Congo) deep-sea fan. *Marine Geology* **176**, 87-99 (2001).

15 Bussmann, I., Dando, P., Niven, S. & Suess, E. Groundwater seepage in the marine environment: role for mass flux and bacterial activity. *Marine ecology. Progress series* **178**, 169-177 (1999).

16 Aharon, P. & Fu, B. Microbial sulfate reduction rates and sulfur and oxygen isotope fractionations at oil and gas seeps in deepwater Gulf of Mexico. *Geochimica et Cosmochimica Acta* **64**, 233-246 (2000).

17 Lein, A. Y. *et al.* Authigenic carbonates in methane seeps from the Norwegian Sea: mineralogy, geochemistry, and genesis. *Lithology and Mineral Resources* **35**, 295-310 (2000).
18 Treude, T. *et al.* Anaerobic oxidation of methane and sulfate reduction along the Chilean continental margin. *Geochimica et Cosmochimica Acta* **69**, 2767-2779, doi:10.1016/j.gca.2005.01.002 (2005).
19 Hinrichs, K.-U. & Boetius, A. in *Ocean Margin Systems* 457-477 (Springer, 2003).
20 Mau, S. *et al.* Estimates of methane output from mud extrusions at the erosive convergent margin off Costa Rica. *Marine Geology* **225**, 129-144, doi:10.1016/j.margeo.2005.09.007 (2006).
21 Treude, T., Boetius, A., Knittel, K., Wallmann, K. & Jørgensen, B. B. Anaerobic oxidation of methane above gas hydrates at Hydrate Ridge, NE Pacific. *Mar. Ecol. Prog. Ser* **264**, 1-14 (2003).
22 Boetius, A., Ferdelman, T. & Lochte, K. Bacterial activity in sediments of the deep Arabian Sea in relation to vertical flux. *Deep Sea Research Part II: Topical Studies in Oceanography* **47**, 2835-2875 (2000).
23 Marlow, J. J. *et al.* Microbial abundance and diversity patterns associated with sediments and carbonates from the methane seep environments of Hydrate Ridge, OR. *Frontiers in Marine Science* **1**, 44 (2014).
24 Bayon, G., Henderson, G. M. & Bohn, M. U–Th stratigraphy of a cold seep carbonate crust. *Chemical Geology* **260**, 47-56, doi:10.1016/j.chemgeo.2008.11.020 (2009).
25 Omoregie, E. O. *et al.* Biogeochemistry and community composition of iron- and sulfur-precipitating microbial mats at the Chefren mud volcano (Nile Deep Sea Fan, Eastern Mediterranean). *Appl Environ Microbiol* **74**, 3198-3215, doi:10.1128/AEM.01751-07 (2008).
26 Aloisi, G., Wallmann, K., Haese, R. R. & Saliège, J. F. Chemical, biological and hydrological controls on the 14C content of cold seep carbonate crusts: numerical modeling and implications for convection at cold seeps. *Chemical Geology* **213**, 359-383, doi:10.1016/j.chemgeo.2004.07.008 (2004).
27 Omoregie, E. O. *et al.* Microbial methane oxidation and sulfate reduction at cold seeps of the deep Eastern Mediterranean Sea. *Marine Geology* **261**, 114-127, doi:10.1016/j.margeo.2009.02.001 (2009).
28 Tang, J., Dietzel, M., Fernandez, A., Tripati, A. K. & Rosenheim, B. E. Evaluation of kinetic effects on clumped isotope fractionation (Δ 47) during inorganic calcite precipitation. *Geochimica et Cosmochimica Acta* **134**, 120-136 (2014).
29 Tripati, A. K. *et al.* Beyond temperature: Clumped isotope signatures in dissolved inorganic carbon species and the influence of solution chemistry on carbonate mineral composition. *Geochimica et Cosmochimica Acta* **166**, 344-371 (2015).
